# Inhibition of Sprouty2 polarizes macrophages toward an M2 phenotype by stimulation with interferon γ and *Porphyromonas gingivalis* lipopolysaccharide

**DOI:** 10.1002/iid3.99

**Published:** 2016-02-26

**Authors:** Ryo Atomura, Terukazu Sanui, Takao Fukuda, Urara Tanaka, Kyosuke Toyoda, Takaharu Taketomi, Kensuke Yamamichi, Hajime Akiyama, Fusanori Nishimura

**Affiliations:** ^1^ Division of Oral RehabilitationDepartment of PeriodontologyFaculty of Dental ScienceKyushu UniversityFukuokaJapan; ^2^ Dental and Oral Medical CenterKurume University School of MedicineFukuokaJapan

**Keywords:** M2 macrophages, periodontal tissue regeneration, *Porphyromonas gingivalis*, Spry2

## Abstract

Periodontitis is a chronic inflammatory disorder caused by specific bacteria residing in the biofilm, particularly *Porphyromonas gingivalis* (*Pg*). Sprouty2 (Spry2) functions as a negative regulator of the fibroblast growth factor (FGF) signaling pathway. We previously demonstrated that sequestration of Spry2 induced proliferation and osteogenesis in osteoblastic cells by basic FGF (bFGF) and epidermal growth factor (EGF) stimulation in vitro, but diminished cell proliferation in gingival epithelial cells. In addition, Spry2 knockdown in combination with bFGF and EGF stimulation increases periodontal ligament cell proliferation and migration accompanied by prevention of osteoblastic differentiation. In this study, we investigated the mechanisms through which Spry2 depletion by interferon (IFN) γ and *Pg* lipopolysaccharide (LPS) stimulation affected the physiology of macrophages in vitro. Transfection of macrophages with Spry2 small‐interfering RNA (siRNA) promoted the expression of genes characteristic of M2 alternative activated macrophages, induced interleukin (IL)‐10 expression, and enhanced arginase activity, even in cells stimulated with IFNγ and *Pg* LPS. In addition, we found that phosphoinositide 3‐kinase (PI3K) and AKT activation by Spry2 downregulation enhanced efferocytosis of apoptotic cells by increasing Rac1 activation and decreasing nuclear factor kappa B (NFκB) p65 phosphorylation but not signal transducer and activator of transcription 1 (STAT1) phosphorylation. Collectively, our results suggested that topical administration of Spry2 inhibitors may efficiently resolve inflammation in periodontal disease as macrophage‐based anti‐inflammatory immunotherapy and may create a suitable environment for periodontal wound healing. These in vitro findings provide a molecular basis for new therapeutic approaches in periodontal tissue regeneration.

## Introduction

Periodontitis is a chronic inflammatory disease that occurs in response to specific bacteria in a biofilm and involves the destruction of tooth‐supporting tissues 1, 2. As lesions associated with chronic periodontitis progress, increasing numbers of macrophages infiltrate into the gingival tissues 3. Macrophages are a first line of defense against invading pathogens, and lipopolysaccharide (LPS) from foreign bacteria induces macrophage activation by the binding with Toll‐like receptors (TLRs) on the surface of macrophages 4. *Porphyromonas gingivalis* (*Pg*) is a key periodontal pathogen associated with both periodontal disease onset and progression 5, 6. LPS from *Pg* activates macrophages through both TLR2 and TRL4,^7^ and specifically, TLR2 activation by *Pg* LPS triggers the downstream stimulation of nuclear factor kappa B (NFκB), leading to the production of pro‐inflammatory cytokines 7, 8, 9, 10.

Macrophages can be categorized into two main distinctly different functional phenotypes. Classical activation of macrophages (M1 macrophages) by stimulation with interferon (IFN) γ or LPS promotes the Th1 response, produces pro‐inflammatory cytokines, kills intercellular pathogens, and initiates adaptive immune responses 11, 12. However, the combination of these responses may cause extensive tissue damage. In contrast, macrophages stimulated by interleukin (IL)‐4 or IL‐13 display a distinct alternative pattern of activation. These so‐called M2 macrophages play roles in Th2 responses, production of anti‐inflammatory cytokines, angiogenesis, scavenging, tissue remodeling, and tissue repair 13, 14, 15, 16. These functional differences are reflected in the expression of the two opposing effector molecules, inducible nitric oxide (iNOS) and arginase. Thus, macrophages are involved in both destruction and regeneration of tissue and play important roles in the interface between inflammation and tissue repair.

Sprouty (Spry) proteins were originally identified in *Drosophila* and interfere with fibroblast growth factor (FGF) signaling by inhibiting the Ras‐Raf1‐mitogen‐activated protein kinase (MAPK) pathway 17, 18. Four human Spry homologs (Spry1–4) have been identified, and Spry2 specifically suppresses the activation of the extracellular signal‐regulated kinase (ERK) pathway in response to various growth factors^19^, ^20^, ^21^, ^22^. Analyses in Spry2‐deficient mice have demonstrated that Spry2 inhibition induces supernumerary teeth in the toothless region of mandible, called the diastema, indicating that Spry2 functions to suppress tooth development in the diastema by blocking FGF signaling 23. In contrast, overexpression of Spry2 in chick embryos results in severely foreshortened frontonasal, maxillary, and mandibular prominences, which causes bilateral facial clefts at the initiation of craniofacial development 24. Intriguingly, suppression of Spry2 and Spry4 enhances murine corneal neovascularization in vivo and improves the recovery of limb perfusion after induction of hind limb ischemia in a mouse model 25. In addition, previous studies in our laboratory have shown that sequestration of Spry2 induces ERK activation, proliferation, and osteogenesis in osteoblastic cells by basic FGF (bFGF) and epidermal growth factor (EGF) stimulation in vitro while suppressing ERK activation and cell proliferation in gingival epithelial cells 26. In addition, we found that Spry2 knockdown combined with bFGF and EGF stimulation increased periodontal ligament cell proliferation and migration, while it prevented osteoblastic differentiation^27^. Therefore, combined application of a Spry2 inhibitor, bFGF, and EGF may effectively facilitate the growth of the periodontal ligament and alveolar bone while preventing tooth ankylosis and blocking gingival epithelial down‐growth toward bony defects. These previous works suggest that Spry2 may have potential applications in periodontal regeneration.

During the onset of periodontal tissue regeneration, the inflammation caused by *Pg* LPS must be resolved, and M2 macrophages play a crucial role in tissue repair. Accordingly, in this study, we investigated the mechanisms through which Spry2 depletion by IFNγ and *Pg* LPS stimulation affected the physiology of macrophages in vitro.

## Materials and Methods

### Cell culture

J774.1 murine macrophage‐like cells were purchased from RIKEN BioResouce Center (Ibaraki, Japan). Cells were cultured in α‐minimal essential medium (α‐MEM) containing 10% fetal bovine serum (FBS), 100 U/ml penicillin, and 100 μg/ml streptomycin at 37°C in an incubator containing 5% CO_2_. Cells were stimulated with 100 ng/ml recombinant murine IFNγ (Peprotech, Rocky Hill, NJ, USA) and 50 ng/ml LPS from *Pg* (Invivogen, San Diego, CA, USA).

### Transfections

J774.1 cells were plated at a density of 3×10^5^ cells/well in 6‐well plates and then transfected with Stealth RNAi duplexes against mouse Spry2 (Invitrogen, Carlsbad, CA, USA), a mixture of three different small‐interfering RNAs (siRNAs; MSS239582, MSS239583, and MSS239584; GC contents: 48%, 52%, and 44%, respectively), using Lipofectamine 2000 Reagent (Invitrogen) according to the supplier's protocol. As a control, we used a Stealth RNAi negative control duplex (Medium GC Duplex; Invitrogen) with a GC content of 48%, suitable for use as a control with Stealth RNAi duplexes of 45–55% GC content. Experimental groups were treated with siRNA at final concentrations of 20 nM in J774.1 cell cultures.

### Total RNA extraction and real‐time polymerase chain reaction (PCR)

The total RNA was extracted from J774.1 cells using ISOGEN II (Nippon Gene, Tokyo, Japan). cDNA was prepared using PrimeScript RT Master Mix (Takara Bio, Shiga, Japan). The PCR primer sequences were as follows: *iNOS*, 5′‐GGACCCAGTGCCCTG CTTT‐3′ (forward) and 5′‐CACCAAGCTCATGCGGCCT‐3′ (reverse); *IL‐6*, 5′‐TGCCTTCTTGGGACTGATG‐3′ (forward) and 5′‐ACTCTGGCTTTGTCTTTCTTGT‐3′ (reverse); *IL‐10*, 5′‐GGTTGCCAAGCCTTATCGTA‐3′ (forward) and 5′‐ACCTGCTCCACTGCCTTGCT‐3′ (reverse); *Ym1*, 5′‐GCAGAAGCTCTCCAGAAGCAATCCTG‐3′ (forward) and 5′‐ATTGGCCTGTCCTTAGCCCAACTG‐3′ (reverse); *bFGF*, 5′‐AGCGGCTCTACTGCAAGAAC‐3′ (forward) and 5′‐TCGTTTCAGTGCCACATACC‐3′ (reverse); *EGF*, 5′‐ACGGTTTGCCTCTTTTCCTT‐3′ (forward) and 5′‐GTTCCAAGCGTTCCTGAGAG‐3′ (reverse); platelet‐derived growth factor (*PDGF*), 5′‐TGATCTCCAACGCCTGCT‐3′ (forward) and 5′‐TCATGTTCAGGTCCAACTCG‐3′ (reverse); vascular endothelial growth factor (*VEGF*), 5′‐CGCCGCAGGAGACAAACCGAT‐3′ (forward) and 5′‐ACCCGTCCATGAGCTCGGCT‐3′ (reverse); and glyceraldehyde‐3‐phosphate dehydrogenase (*Gapdh*), 5′‐GGTCGGAGTCAACGGATTTG‐3′ (forward) and 5′‐ATGAGCCCCAGCCTTCTCCAT‐3′ (reverse). PCR was performed using SYBR Green II (Takara Bio) on an Applied Biosystems StepOnePlus Real‐Time System (Life Technologies) under the following conditions: 95°C for 10 sec, followed by 40 cycles of 95°C for 15 sec and 60°C for 60 sec. *Gapdh* was used as an internal control. The expression of the target genes was calculated using the ΔΔC_T_ method.

### Measurement of cytokine production

J774.1 cells transfected with Spry2 siRNA or control siRNA were stimulated with IFNγ and *Pg* LPS for 24 h. The levels of cytokines (IL‐12, IL‐6, tumor necrosis factor [TNF]‐α, and IL‐10) in the supernatants were determined using Quantikine ELISA Kit (R&D Systems, Minneapolis, MN, USA) according to the manufacturer's instructions. Each assay was performed in triplicate and averaged.

### Western blot analysis

J774.1 cells transfected with control siRNA or Spry2 siRNA were lysed and analyzed using western blotting. Proteins were separated on polyacrylamide gels, transferred to membranes, and detected using anti‐mouse Spry2 (Upstate, Lake Placid, NY, USA), anti‐iNOS (BD Pharmingen, San Jose, CA, USA), anti‐arginase 1 (Arg1), anti‐inhibitor kappa B‐α (IκB‐α; Santa Cruz Biotechnology, Santa Cruz, CA, USA), anti‐phospho‐NFκB p65 (p‐p65; Ser536), anti‐NFκB p65, anti‐phospho‐AKT (p‐AKT; Ser473), anti‐AKT, anti‐phospho‐phosphoinositide 3‐kinase (p‐PI3K) p85 (Tyr458)/p55 (Tyr199), anti‐PI3K p85 (Cell Signaling Technology, Danvers, MA, USA), anti‐phospho‐signal transducer and activator of transcription 1 (p‐STAT1; Tyr701; Abcam, Cambridge, UK), or anti‐STAT1 (Cell Signaling Technology) antibodies. As a control, β‐actin was detected using anti‐β‐actin antibodies (Cell Signaling). Immunoreactive proteins were visualized using ECL Western Blotting Substrate (Pierce, Rockford, IL, USA), and the membranes were then exposed using an Image Quant LAS4000 (GE Healthcare, Tokyo, Japan). Densitometric analysis of bands was performed using ImageJ (National Institutes of Health, Bethesda, MD, USA).

### Arginase activity assay

Transfected J774.1 cells were lysed for 10 min in 100 μl of 10 mM Tris‐HCl (pH 7.4) containing 1 μM pepstatin A, 1 μM leupeptin, and 0.4% (w/v) Triton X‐100. Supernatants were used for arginase activity assays. The activity of intracellular arginase was determined using a QuantiChrom Arginase Assay Kit (BioAssay Systems, Hayward, CA, USA).

### Measurement of the efferocytosis of apoptotic cells

Murine apoptotic thymocytes were induced by exposure to UV irradiation at 312 nm for 10 min. Transfected J774.1 cells treated with IFNγ and LPS and apoptotic cells stained with propidium iodide (Cell Signaling Technology) were co‐cultured (1:10) for 60 min and then extensively washed with phosphate‐buffered saline (PBS), fixed with 4% paraformaldehyde, permeabilized with 0.5% Triton X‐100, and blocked with 3% bovine serum albumin in PBS. Actin was stained with Alexa Flour 488‐conjugated phalloidin (Invitrogen Life Technologies). The coverslips were mounted using PermaFluor Mounting medium (Thermo Fisher Scientific, Miami, FL, USA). For confocal immunofluorescence analysis, cells were visualized with a Zeiss LSM 700 confocal microscope using Zen software.

### Rac1 activity assay

Rac1 activation was evaluated by pull‐down assay using a Rac1 Activation Assay Kit (Cell Biolabs, San Diego, CA, USA). Extracts containing equal amounts of protein were incubated in microcentrifuge tubes with PAK1 PBD (p21‐binding domain of human p21 activating kinase‐1) agarose beads at 4°C for 1 h with gentle agitation. Active Rac1 bound to agarose beads was pelleted by centrifugation. Beads were washed and non‐specifically bound proteins were removed. The bead pellet was suspended in sodium dodecyl sulfate polyacrylamide gel electrophoresis sample buffer and subjected to western immunoblotting, followed by probing with anti‐Rac1 antibodies.

### Statistical analysis

Experimental data were expressed as means ± standard deviations (SDs). All experiments were performed in triplicate, and at least three independent experiments were repeated. Statistical analyses were determined by unpaired Student's *t*‐tests or one‐way analysis of variance (ANOVA). Differences with *P* values of less than 0.05 were considered significant.

## Results

### **Spry2** knockdown shifted cellular marker expression from the M1 to M2 phenotype in J774.1 macrophages stimulated with IFNγ and *Pg* LPS

**Figure 1:**
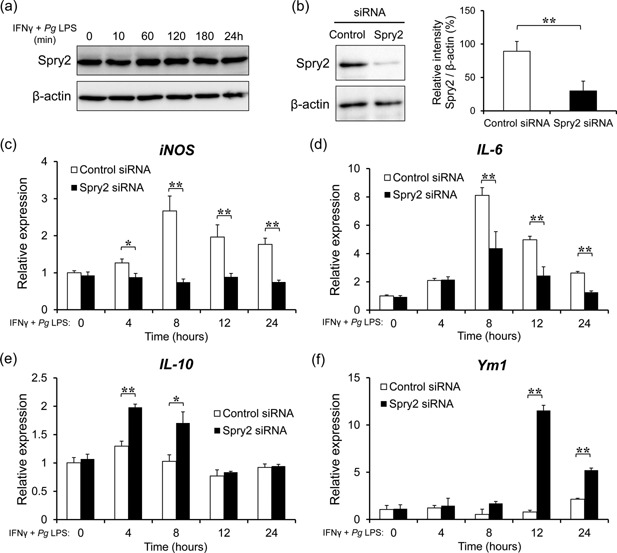
Spry2 knockdown shifted cellular marker expression from the M1 to M2 phenotype in J774.1 macrophages stimulated with IFNγ and *Porphyromonas gingivalis* LPS. (a) J774.1 cells were cultured with 100 ng/ml IFNγ and 50 ng/ml *Porphyromonas gingivalis* (*Pg*) LPS for the indicated times. Cell extracts were resolved by SDS‐PAGE and immunoblotted with anti‐Spry2 antibodies. As a control, β‐actin was detected using anti‐β‐actin antibodies. (b) Representative western blot analysis of Spry2 expression in control siRNA‐ or Spry2 siRNA‐transfected J774.1 cells. Quantification of the ratio of Spry2 protein levels relative to that of β‐actin was carried out using ImageJ. The significance of differences between groups was determined by two‐tailed unpaired Student's *t*‐tests: *P < 0.05; **P < 0.01. Data represent means ± SDs; n = 3. Similar results were obtained in three independent experiments. (c–f) Transfected J774.1 cells were cultured in the presence of IFNγ and *Pg* LPS for the indicated times and were subjected to total RNA extraction, and mRNA levels of (c) *iNOS*, (d) *IL‐6*, (e) *IL‐10*, and (f) *Ym1* were determined using quantitative real‐time PCR. *Gapdh* was used as a control. The significance of differences between groups was determined by two‐tailed unpaired Student's *t*‐tests: *P < 0.05; **P < 0.01. Data represent means ± SDs; n = 3. Similar results were obtained in three independent experiments.

To investigate whether Spry2 functioned downstream of the TLR signal pathway, we first examined Spry2 protein levels in J774.1 macrophages stimulated with IFNγ and *Pg* LPS. As shown in Figure 1a, Spry2 was constitutively expressed in J774.1 cells, regardless of the addition of IFNγ and *Pg* LPS. Next, to evaluate the physiological activity of Spry2 in macrophages, J774.1 cells were transfected with Spry2 siRNA and then stimulated with IFNγ and *Pg* LPS. Spry2 siRNA efficiently suppressed the expression of endogenous Spry2, as measured by western blot analysis (Fig. 1b). mRNA expression levels of M1 and M2 macrophage cellular marker genes were measured using quantitative real‐time RCR at 4, 8, 12, and 24 h after stimulation of macrophages by IFNγ and *Pg* LPS. Transfection with Spry2 siRNA decreased the expression of genes encoding the M1 markers iNOS and IL‐6 (Fig. 1c and d). In contrast, the expression of M2 marker genes (*IL‐10* and *Ym1*) was higher in Spry2 siRNA‐transfected cells than in control siRNA‐transfected cells (Fig. 1e and f).

### Inhibition of Spry2 reduced pro‐inflammatory cytokine production and promoted anti‐inflammatory cytokine production in J774.1 cells stimulated with IFNγ and *Pg* LPS

**Figure 2:**
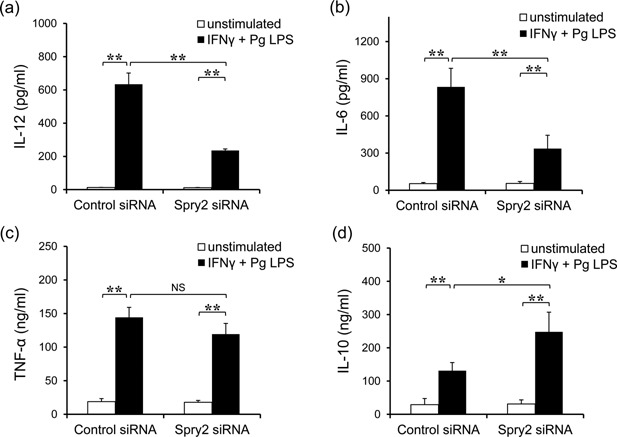
Inhibition of Spry2 reduced pro‐inflammatory cytokine production and promoted anti‐inflammatory cytokine production in J774.1 cells stimulated with IFNγ and *Pg* LPS. Transfected J774.1 cells were cultured in the presence of IFNγ and *Pg* LPS for 24 h, and the levels of (a) IL‐12, (b) IL‐6, (c) TNF‐α, and (d) IL‐10 were measured from supernatants. The significance of differences between groups was determined by one‐way ANOVA/Tukey's test: *P < 0.05; **P < 0.01. Data represent means ± SDs; n = 3. Similar results were obtained in three independent experiments.

Spry2 siRNA‐transfected J774.1 macrophages were stimulated with IFNγ and *Pg* LPS, and culture supernatants were analyzed for pro‐inflammatory and anti‐inflammatory cytokines. The concentrations of the pro‐inflammatory cytokines IL‐12 and IL‐6 were significantly reduced in Spry2 siRNA‐transfected cells in the presence of IFNγ and *Pg* LPS (Fig. 2a and b). In contrast, the concentration of the anti‐inflammatory cytokine IL‐10 was enhanced by induction with Spry2 siRNA (Fig. 2c). Production of the pro‐inflammatory molecule TNF‐α was slightly lower, but the difference was not significant (Fig. 2c). These results were consistent with the altered macrophage phenotype and showed that Spry2 siRNA could alter cytokine production in macrophages stimulated with IFNγ and *Pg* LPS to reduce the inflammatory status.

### Spry2 depletion decreased iNOS activity and increased arginase activity in J774.1 cells stimulated with IFNγ and *Pg* LPS

**Figure 3:**
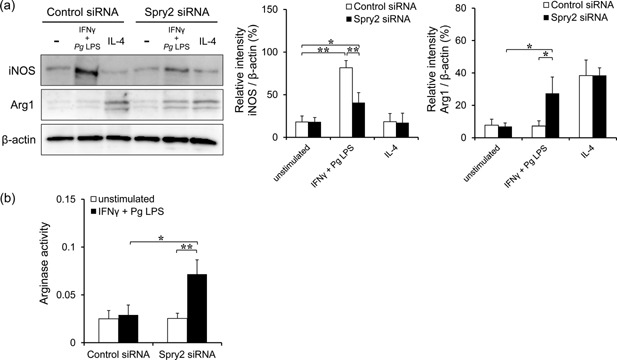
Spry2 depletion decreased iNOS activity and increased arginase activity in J774.1 cells stimulated with IFNγ and *Pg* LPS. (a) Representative western blots of iNOS and arginase expression in control siRNA‐ or Spry2 siRNA‐transfected J774.1 cells. Quantification of the ratio of iNOS and Arg1 protein levels relative to that of β‐actin was carried out using ImageJ. The significance of differences between groups was determined by one‐way ANOVA/Tukey's test: *P < 0.05; **P < 0.01. Data represent means ± SDs; n = 3. Similar results were obtained in three independent experiments. (b) The arginase activity of J774.1 cells stimulated with IFNγ and *Pg* LPS. J774.1 cells were treated with or without IFNγ and *Pg* LPS for 24 h after pretreatment with control siRNA or Spry2 siRNA. Activity was determined by measurement of the conversion of arginine into urea. The significance of differences between groups was determined by one‐way ANOVA/Tukey's test: *P < 0.05; **P < 0.01. Data represent means ± SDs; n = 3. Similar results were obtained in three independent experiments.

iNOS is activated in response to inflammatory stimuli as macrophages shift toward the M1 phenotype, whereas the expression of arginase is induced by Th2‐type cytokines, and the balance between iNOS and arginase is correlated with Th1 and Th2 responses 28. Therefore, iNOS and arginase protein expression levels in Spry2 siRNA‐transfected macrophages treated with IFNγ and *Pg* LPS were measured by western blotting. iNOS protein expression was detectable in control cells, but was significantly reduced in Spry2 siRNA‐transfected J774.1 cells exposed to IFNγ and *Pg* LPS (Fig. 3a). In contrast, Arg1 protein expression was substantially enhanced when Spry2 siRNA‐transfected macrophages were stimulated with IFNγ and *Pg* LPS (Fig. 3a). Consistent with this result, the addition of IFNγ and *Pg* LPS to Spry2‐knockdown cells resulted in increased arginase activity (Fig. 3b).

### Inhibition of Spry2 enhanced the expression of genes encoding growth factors in J774.1 cells stimulated with IFNγ and *Pg* LPS

**Figure 4:**
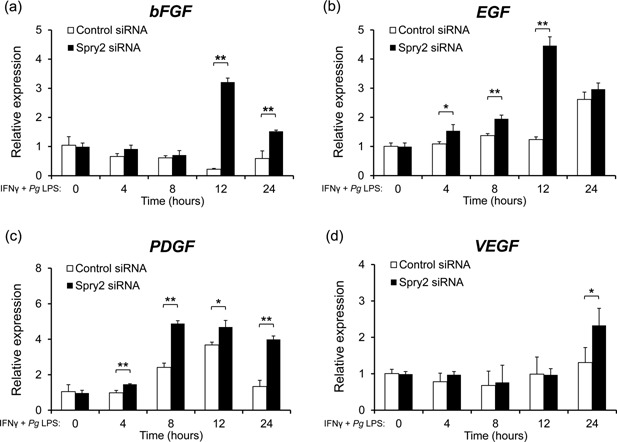
Inhibition of Spry2 enhanced the expression of genes encoding growth factors in J774.1 cells stimulated with IFNγ and *Pg* LPS. (a–d) Transfected J774.1 cells were cultured in the presence of IFNγ and *Pg* LPS for the indicated times and subjected to total RNA extraction. The expression levels of *bFGF*, *EGF*, *PDGF*, and *VEGF* mRNAs were then determined by quantitative real‐time PCR. Similar results were obtained in three independent experiments. *Gapdh* was used as a control. The significance of differences between groups was determined by two‐tailed unpaired Student's *t*‐tests: *P < 0.05; **P < 0.01. Data represent means ± SDs; n = 3. Similar results were obtained in three independent experiments.

M2 macrophages are thought to play a central role in the proliferative response of various cell types associated with wound healing, and prior studies have indicated that these cells produce growth factors, such as bFGF and EGF 29. The expression levels of genes encoding growth factor were measured using quantitative real‐time RCR at 4, 8, 12, and 24 h after stimulation of J774.1 macrophages with IFNγ and *Pg* LPS. As expected, we observed higher expression of *bFGF*, *EGF*, *PDGF*, and *VEGF* mRNAs in Spry2 siRNA‐transfected cells (Fig. 4a–d).

### Spry2 suppression enhanced efferocytosis of apoptotic cells by inducing Rac1 activation when J774.1 cells were stimulated with IFNγ and *Pg* LPS

**Figure 5:**
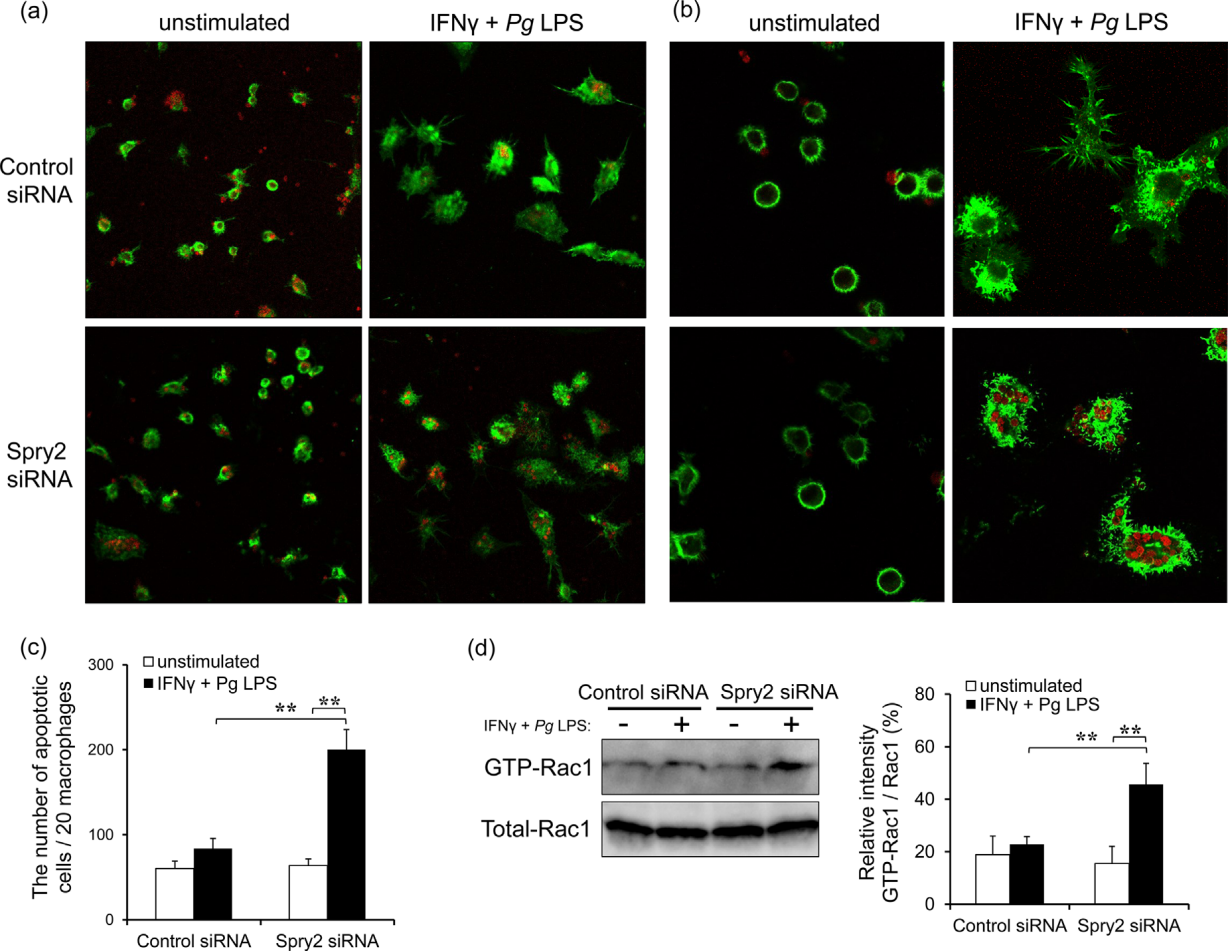
Spry2 suppression enhanced efferocytosis of apoptotic cells by inducing Rac1 activation when J774.1 cells were stimulated with IFNγ and *Pg* LPS. (a, b) For efferocytosis assays, transfected J774.1 cells were treated with IFNγ and *Pg* LPS, and mouse apoptotic thymocytes were stained with propidium iodide (red). These cells were co‐cultured (1:10), and F‐actin expression in J774.1 cells was visualized by phalloidin (green). The images shown are at (a) 200× or (b) 400× magnification, and represent one of three similar experiments. (c) The number of apoptotic cells effferocytosed by 20 macrophages was counted. The significance of differences between groups was determined by one‐way ANOVA/Tukey's test: *P < 0.05; **P < 0.01. Data represent means ± SDs; n = 3. Similar results were obtained in three independent experiments. (d) The activation of Rac1 was monitored by pulling down the active G proteins with the substrate binding domain of PAK1 as a GST fusion protein. The total amounts of Rac1 in the cell lysates used for pull‐down of active G proteins were monitored by western blotting with the appropriates antibodies. Representative experiments are shown. Quantification of GST‐Rac1 levels relative to total Rac1 was carried out using ImageJ. The significance of differences between groups was determined by one‐way ANOVA/Tukey's test: *P < 0.05; **P < 0.01. Data represent means ± SDs; n = 3. Similar results were obtained in three independent experiments.

Efferocytosis is an essential process involved in removal or clearance of apoptotic cells, and this process is crucial for maintenance of normal tissue homeostasis, embryoligic development, and resolution of inflammation 30, 31. Macrophages produce anti‐inflammatory cytokines after recognizing, engulfing, and internalizing apoptotic cells. To evaluate efferocytosis of apoptotic cells by macrophages, Spry2 siRNA‐transfected J774.1 macrophages stimulated with IFNγ and *Pg* LPS were co‐cultured with murine apoptotic thymocytes. Control siRNA‐transfected macrophages were transformed into huge and dendri‐form cells by following stimulation with IFNγ and *Pg* LPS. However, efferocytosis was not observed in these control cells. On the other hand, efferocytosis of apoptotic cells was markedly enhanced in Spry2 siRNA‐transfected macrophages stimulated with IFNγ and *Pg* LPS (Fig. 5a–c).

The engulfment of apoptotic cells relies on the signaling of Rho family GTPases, specifically Rac1, and subsequent cytoskeletal reorganization 31, 32, 33. Consistent with the increase in the number of apoptotic cells engulfed by macrophages (Fig. 5c), the activation of Rac1 was markedly increased in Spry2 siRNA‐transfected J774.1 cells, resulting in induction of efferocytosis in apoptotic cells (Fig. 5d). These findings indicated that Spry2 inhibition promoted efferocytosis in apoptotic cells via Rac1 activation in macrophages after stimulation with IFNγ and *Pg* LPS.

### Spry2 inhibition down‐regulated *Pg* LPS‐induced NFκB activation but not IFNγ‐induced STAT1 activation in J774.1 macrophages by increasing AKT and PI3K activation

**Figure 6:**
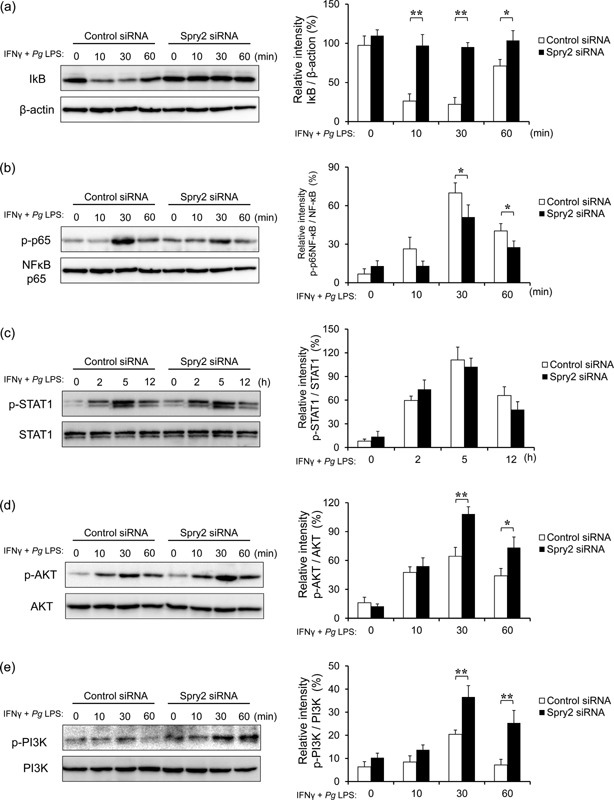
Spry2 inhibition downregulated *Pg* LPS‐induced NFκB activation but not IFNγ‐induced STAT1 activation in J774.1 macrophages by increasing AKT and PI3K activation. After stimulation by IFNγ and *Pg* LPS for the indicated times, whole cell lysates prepared from J774.1 cells transfected with Spry2‐ or control‐siRNA were immunoblotted with anti‐IκB antibodies. β‐actin was used as a control. Protein expression was quantified using the ImageJ program. Representative western blots of (a) IκB degradation, and phosphorylated (b) NFκB p65, (c) STAT1, (d) AKT, and (e) PI3K in siRNA‐transfected J774.1 cells stimulated with IFNγ and *Pg* LPS for the indicated times. Quantification of phosphorylated protein levels relative to total protein was carried out using ImageJ. The significance of differences between groups was determined by two‐tailed unpaired Student's *t*‐tests: *P < 0.05; **P < 0.01. Data represent means ± SDs; n = 3. Similar results were obtained in three independent experiments.

The TLR signaling pathway regulates the transcription factor NFκB in LPS‐stimulated macrophages. Ubiquitination of IκB leads to its proteasomal degradation and phosphorylation of NFκB p65 34. To investigate the mechanisms through which Spry2 inhibition affects TLR and IFNγ receptor signaling pathways in macrophages, we next examined IκB degradation and NFκB p65 phosphorylation in Spry2 siRNA cells. As shown in Figure 6a, IκB was degraded after 10 min of *Pg* LPS and IFNγ stimulation in control cells. However, IκB degradation was not observed in Spry2 siRNA‐transfected J774.1 macrophages. Consistent with this result, lower levels of phosphorylated NFκB p65 were observed in *Pg* LPS‐ and IFNγ‐stimulated Spry2 siRNA macrophages compared with that in control cells (Fig. 6b).

IFNγ mediates iNOS upregulation via the Janus kinase (JAK)/ STAT pathway. STAT1 is phosphorylated on tyrosine 701 following stimulation with IFNγ. Therefore, we explored the effects of Spry2 inhibition on STAT1 phosphorylation in response to IFNγ and *Pg* LPS. Western blot analysis showed that phosphorylation of STAT1 in Spry2 siRNA‐transfected cells was not different from that in the control cells (Fig. 6c).

The activation of PI3K and its downstream targets, including AKT, by stimulation with growth factors suppresses LPS‐induced NFκB signaling cascades in macrophages, resulting in decreased production of pro‐inflammatory cytokines and nitric oxide 35. Therefore, to identify the possible causative mechanism, we examined PI3K and AKT activation after IFNγ and *Pg* LPS stimulation in Spry2‐transfected J774.1 cells by western blotting. Spry2 siRNA increased the levels of both PI3K and AKT phosphorylation in J774.1 cells stimulated with IFNγ and *Pg* LPS (Fig. 6d and e). These data suggested that Spry2 inhibition promoted phosphorylation of PI3K and AKT, thereby reducing NFκB activation but not STAT1 following stimulation with IFNγ and *Pg* LPS.

## Discussion

In this study, we identified a novel role for Spry2 protein in the regulation of macrophage polarization in vitro. Suppression of Spry2 in macrophages promoted the expression of genes and cytokines that characterize M2 macrophages, when the cells were stimulated with IFNγ and *Pg* LPS. Our analysis showed that PI3K and AKT activation by Spry2 downregulation enhanced efferocytosis of apoptotic cells by promoting Rac1 activation and inhibited *Pg* LPS‐induced NFκB activation.

**Figure 7:**
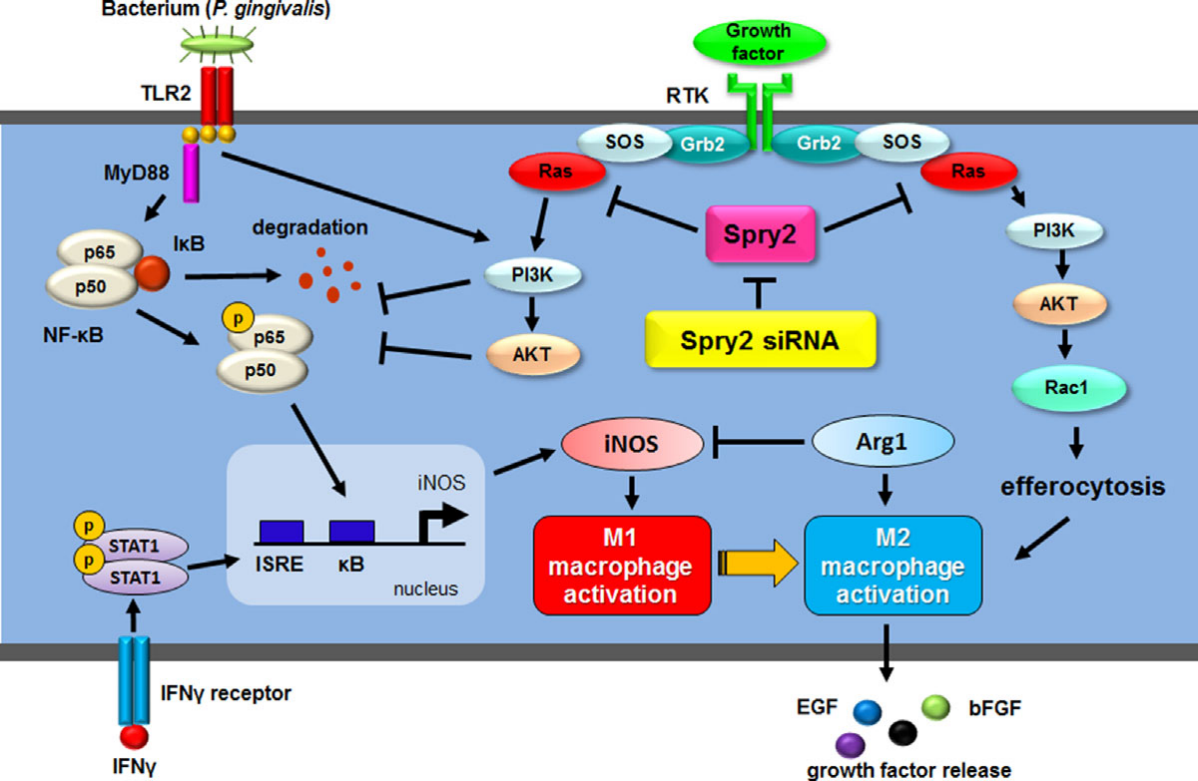
Proposed model of the involvement of Spry2 and Spry2 siRNA in TLR, IFNγ receptor, and growth factor signaling pathways for suppression of M1 polarization and enhancement of M2 polarization in macrophages. Upon *Pg* LPS and IFNγ binding to their respective receptors and activating the respective signal pathways, Spry2 becomes activated and interacts with various components of the PI3K/AKT signaling pathway. Spry2 siRNA inhibits endogenous Spry2, thereby promoting growth factor‐induced Rac1 activation. Spry2 siRNA enhances the efferocytosis of apoptotic cells, leading to polarization into M2 macrophage activation. *Pg* LPS stimulation increases IκB degradation and NFκB p65 phosphorylation, and IFNγ stimulation promotes STAT1 phosphorylation. Stimulation with both IFNγ and LPS shifts macrophages toward the M1 phenotype by activating iNOS expression. PI3K and AKT activation by Spry2 siRNA interferes with IκB degradation and NFκB p65 phosphorylation, resulting in suppression of M1 polarization. Polarized M2 macrophages release various growth factors. See the Discussion for a more in‐depth explanation. MyD88, myeloid differentiation primary response 88. ISRE, interferon‐stimulated response element.

Spry2 is a regulator of receptor tyrosine kinase (RTK) signaling and inhibits ERK activity through regulation of Ras by interfering with growth factor receptor‐bound protein 2 (Grb2) and sons of sevenless (Sos) binding 36, 37. The interaction between Grb2 and RTK is prevented and RTK signaling is inhibited as a result of the interaction between Spry2 and Grb2 21, 38. In addition to the Ras/ERK pathway, Spry2 can also suppress the PI3K/AKT signaling pathway by promoting the activation and stability of phosphatase and tensin homolog (PTEN) 39, 40, 41. In contrast, loss of Spry2 protein results in hyper‐activation of PI3K/AKT signaling to drive cell proliferation and invasion 42. PI3K and AKT phosphorylate p21‐activated kinase (PAK), providing a target for the small GTP‐binding protein Rac1 43, 44. Spry2 has been reported to induce anti‐migratory effects by inhibiting Rac1 activation 45. The importance of Rho GTPases in efferocytosis has been demonstrated in many studies 46, 47, and Rac1 activation is important in efferocytosis through modification of the cytoskeleton, lamellipodia formation, and membrane ruffle formation. In contrast, RhoA activation inhibits this process 48. Several studies have shown that the engulfment of apoptotic cells by efferocytosis can result in production of anti‐inflammatory mediators, including transforming growth factor beta (TGFβ) and IL‐10 49, 50. In addition, recognition of apoptotic cells by macrophages during efferocytosis activates the phosphorylation of STAT3, an enhancer of anti‐inflammatory signaling, leading to a polarization of macrophages into the M2 phenotype with increased expression of M2 markers as well as M2‐related cytokines and growth factors 51, 52. Therefore, our results suggested that the sequestration of Spry2 increased PI3K/AKT phosphorylation and Rac1 activation, thereby promoting efferocytosis of apoptotic cells and shifting macrophages toward the M2 phenotype (Fig. 7).

PI3K/AKT signaling can also stimulate LPS‐induced TLR signaling and subsequently increase the anti‐inflammatory response 35. The PI3K/AKT pathway is activated by TLR signaling in a feedback loop that inhibits the response to TLR activators. That is, growth factors can enhance PI3K and AKT activation and contribute to M2 polarization through a mechanism independent of the IL‐4/IL‐13 signaling pathway 35. Furthermore, PI3K negatively regulates TLR signaling during exposure to pathogens, and the promotion of PI3K activation leads to decreased IL‐12 and enhancement of IL‐4 and IL‐10 production in antigen‐presenting cells 53. In this study, we showed that PI3K/AKT activated by Spry2 siRNA suppressed IκB degradation and NFκB p65 phosphorylation but not STAT1 phosphorylation. This crosstalk between TLR and growth factor signaling resulted in decreased iNOS expression and interference with the shift toward M1 macrophages (Fig. 7). However, as shown in Figure 2c, the differences in TNF‐α production between control siRNA‐ and Spry2 siRNA‐transfected macrophages in the presence of IFNγ and *Pg* LPS were not significant. The expression of TNF‐α is mediated by the transcription factor LPS‐induced TNF‐α factor (LITAF) in an LPS‐induced process 54. LITAF is an important mediator of the LPS‐induced inflammatory response that can be distinguished from the NFκB pathway 55. Therefore, normal production of TNF‐α by Spry2 suppression with IFNγ and *Pg* LPS co‐stimulation may be caused by LITAF signaling but not NFκB signaling.

Interestingly, Spry2 and Spry4 are physiologically critical negative regulators of angiogenesis in vivo, and inhibition of their activities may be a new therapeutic target for treating peripheral ischemic diseases 25. In addition, the downregulation of Spry2 promotes cell proliferation, differentiation, and migration of endothelial cells, thereby promoting angiogenesis and inducing skin wound healing in mice 56. Moreover, the suppression of Spry2 aggressively increases elongation of axon growth of sensory neurons 57. In our previous studies, we observed that Spry2 downregulation promoted the phosphorylation of ERK and the proliferation of osteoblastic cells after bFGF and EGF stimulation. Moreover, Spry2 downregulation increased osteogenesis by upregulating Ras‐responsive Runx2 expression through the induction of Twist, a negative regulator of Runx2. In contrast, suppression of Spry2 expression enhanced ubiquitination and degradation of EGF receptors, resulting in decreased proliferation of gingival epithelial cells 26. In addition, we also found that Spry2 siRNA promoted lamellipodia formation induced by the stimulation of bFGF and EGF via AKT/PI3K and Rac1 activation in PDL cells, thereby activating cell migration while suppressing osteoblastic differentiation 27. Thus, Spry2 downregulation could benefit periodontal tissue regeneration by increasing the stimulation of growth factors. The results presented in this study demonstrated that M2 macrophages induced by Spry2 inhibition may enhance the production of growth factors such as bFGF and EGF and involve in the functions of osteoblasts, gingival epithelial cells, and periodontal ligament cells, which take up these cytokines. Therefore, our data suggested that Spry2 suppression may yield a favorable environment for periodontal regeneration even without bFGF and EGF application.

In summary, our results suggested that topical administration of Spry2 inhibitors may efficiently resolve inflammation in periodontal lesion as macrophage‐based anti‐inflammatory immunotherapy and may create a suitable environment for periodontal wound healing. These in vitro findings provide a molecular basis for new therapeutic approaches in periodontal tissue regeneration.
